# Lumbar spine intrathecal transplantation of neural precursor cells promotes oligodendrocyte proliferation in hot spots of chronic demyelination

**DOI:** 10.1111/bpa.13040

**Published:** 2021-11-29

**Authors:** Paschalis Theotokis, Evangelia Kesidou, Dimitra Mitsiadou, Steven Petratos, Olympia Damianidou, Marina Boziki, Anastasia Chatzidimitriou, Nikolaos Grigoriadis

**Affiliations:** ^1^ Laboratory of Experimental Neurology and Neuroimmunology Second Department of Neurology AHEPA University Hospital Thessaloniki Greece; ^2^ Department of Neuroscience Central Clinical School Monash University Prahran Victoria Australia; ^3^ Institute of Applied Biosciences Centre for Research and Technology Hellas Thessaloniki Greece

**Keywords:** connexins, EAE, g‐ratio, intrathecal transplantation, NPCs, oligodendrogenesis

## Abstract

Experimental autoimmune encephalomyelitis (EAE) is a basic and reliable model used to study clinical and pathological hallmarks of multiple sclerosis (MS) in rodents. Several studies suggest neural precursor cells (NPCs) as a significant research tool while reporting that transplanted NPCs are a promising therapeutic approach to treating neurological disorders, such as MS. The main objective was to approach a preclinical, in vivo scenario of oligodendrogenesis with NPCs, targeting the main chronic demyelinated lumbosacral milieu of EAE, via the least invasive delivery method which is lumbar puncture. We utilized MOG_35‐55_ peptide to induce EAE in C57BL/6 mice and prior to the acute relapse, we intervened with either the traceable GFP^+^ cellular therapy or saline solution in the intrathecal space of their lumbar spine. A BrdU injection, which enabled us to monitor endogenous proliferation, marked the endpoint 50 days post‐induction (50 dpi). Neuropathology with high‐throughput, triple immunofluorescent, and transmission electron microscopy (TEM) data were extracted and analyzed. The experimental treatment attenuated the chronic phase of EAE (50 dpi; score <1) following an acute, clinical relapse. Myelination and axonal integrity were rescued in the NPC‐treated animals along with suppressed immune populations. The differentiation profile of the exogenous NPCs and endogenous BrdU^+^ cells was location‐dependent where GFP^+^‐rich areas drove undifferentiated phenotypes toward the oligodendrocyte lineage. In situ oligodendrocyte enrichment was demonstrated through increased (*p* < 0.001) gap junction channels of Cx32 and Cx47, reliable markers for proliferative oligodendroglia syncytium. TEM morphometric analysis ultimately manifested an increased g‐ratio in lumbosacral fibers of the recovered animals (*p* < 0.001). Herein, we suggest that a single, lumbar intrathecal administration of NPCs capacitated a viable cellular load and resulted in clinical and pathological amelioration, stimulating resident OPCs to overcome the remyelination failure in EAE demyelinating locale.

## Graphical abstract

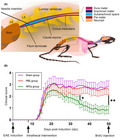

## 1 INTRODUCTION

A corpus of evidence exists supporting the therapeutic efficacy of neural precursor cells (NPCs) for various neurological diseases [1, 2]. These multipotent stem cells are capable of proliferating and they constitute, among others, the source of myelin‐forming cells, such as oligodendrocyte progenitors, olfactory nerve ensheathing cells, and Schwann cells, which are of great importance in demyelinating diseases like multiple sclerosis (MS) [3, 4]. As such, NPCs have been the focus of many studies investigating their efficiency at ameliorating experimental autoimmune encephalomyelitis (EAE), potentiated mainly through neurorepair, advocating as a plausible clinically relevant experimental model of MS [5].

Considering that endogenous cells within the brains of individuals living with MS have already limited reparative capacity in chronic lesions, there is inevitably a substantial failure in myelin regeneration of axons by the existing oligodendrocytes [6, 7]. In this context, transplantation of NPCs can offer an important advantage by controlling the exacerbated immune reactions through their immunomodulatory effect during CNS inflammation [8, 9] and by providing a trophic support through chemical coupling with the host tissue [7, 10, 11]. This has been shown in the demyelinating cuprizone model where intracerebroventricularly (ICV) transplanted NPCs, were shown to migrate in the nearby corpus callosum (cc) providing trophic factors on resident oligodendrocyte precursor cells (OPCs) and thereby aiding to overcome the remyelination failure [12].

One aspect for the remyelinating process to begin, requires the NPCs to migrate close to the lesioned areas, proliferate successfully, and differentiate into remyelinating oligodendrocytes [13]. However, in multifocal neuroinflammatory diseases such as MS, restoration of myelin and prevention of secondary degeneration would require a sufficient dissemination of cells, because resident OPCs closer to the lesions are not always capable of activating their regenerating properties and they remain in a quiescent state instead [14]. Intravenous, intraocular (retro‐orbital) or transplantation around brain ventricular areas (ICV and intracisternal administration) have shown overall positive effects on clinical amelioration of EAE [8, 15, 16, 17, 18, 19] although the proposed mechanism of action has been attributed to peripheral immunomodulation or cytokine production, since the majority of EAE lesions are located in lower spinal cord myelinated fascicles [20, 21].

Previous collaborations of our laboratory, have validated the in vitro growth [22] and pathotropism effect in a cytokine‐based NPC–OPC coculture environment [12]. The same research group verified that ICV‐transplanted NPCs, survived at least one month in the growth factor‐poor environment of naïve mice and migrated only in response to EAE [22]. In the current study, we utilized lumbar puncture, a routinely applicable clinical technique, as a route to disseminate the NPCs neuroanatomically closer to the region of interest and study any beneficial, in situ effect they employ over chronic inflammatory demyelination. Furthermore, we studied the pathotropism‐driven migration and differentiation of those cells. Lastly, we quantified the long‐term sequelae they exert over local OPCs, their proliferation, and the ability to conjointly orchestrate the remyelinating process.

## 2 MATERIALS AND METHODS

### 2.1 Animal handling and ethics

C57BL/6 female mice (*N* = 30) were purchased from the Hellenic Pasteur Institute and maintained at the pathogen‐free animal facility (SPF; Biosafety level 3) of AHEPA University Hospital (EL 54 BIO29). The treatment of animals in all procedures was conducted in strict accordance with Institutional guidelines (Greek Regulations) set by the European Communities Council Directive of November 24, 1986 (86/609/EEC).

### 2.2 EAE induction and scoring

Six‐ to eight‐week‐old C57BL/6 mice (*N* = 28) were immunologically challenged by 200 μg subcutaneous (s.c) injection of myelin oligodendrocyte glycoprotein 35–55 peptide (MOG_35‐55_; MEVGWYRSPFSRVVHLYRNGK) in incomplete Freund's adjuvant (IFA) containing 4 mg/ml of Mycobacterium tuberculosis and developed chronic EAE [23]. Mice were also intraperitoneally (i.p.) injected with 200 ng pertussis toxin (PTX), followed by a second booster dose at 2 days post‐induction (2 dpi). A booster dose of MOG/CFA was also administered at 7 dpi. Animals were assessed on a daily basis for any clinical EAE symptoms and their weight was also recorded. Scoring criteria were as follows: 0, clinically healthy; 1, limpness in tail; 1.5, wobbling gait; 2, light hind limb ataxia; 2.5, evident hind limb paralysis; 3, complete hind limb paralysis and difficulty in recumbency; 3.5, ascending prostration of forelimbs; 4, quadriplegia or paraplegia with incontinence; 4.5, complete forelimb paralysis exhibiting moribund state; 5, death.

### 2.3 Postnatal NPCs cultures

Mouse NPCs were obtained from dissected cortex of newborn transgenic C57BL/6 mice expressing green fluorescent protein C57BL/6‐TgN(ACTbEGFP)1Osb (Jackson Lab, strain 003291) and developed in neurospheres, as previously described [10]. For neurosphere phenotyping and validation of stem cell multipotency, NPCs were cultured in DMEM/F‐12 basal medium (21331‐020, Gibco) and their differentiation pattern was evaluated, as extensively been described by our laboratory in the past [24, 25]. 5000 neurospheres or 1 x 10^6^ cells were re‐suspended in 10 μl PBS and were ready for intrathecal delivery.

### 2.4 Lumbar intrathecal transplantation

A total volume of 10 μl (containing 1 × 10^6^ NPCs) were administered in the lumbosacral subarachnoid cistern of the spinal cord of the main experimental group (*N* = 13). This cell delivery paradigm was accompanied by a placebo group receiving only PBS solution (*N* = 10). According to experimental requirements, a sham intervention should also be performed as an additional control, thus, a third, control group was also included that was subjected to an empty needle (*N* = 5). Eight days after the induction of EAE, animals were weighted, randomized into treatment groups, and anesthetized with i.p. injection of 12.5% ketamine and 6.25% xylazine in 0.9% saline solution (80–100 mg/kg). Mice were immobilized on an operating stage under a stereomicroscope in a flexed position that facilitated the penetration of the lower lumbar area of the spine. After sterilization with ethanol, a small skin incision was made at the lumbar level revealing the spinous processes of the vertebral column in order to identify the L5 and L6 vertebrae. A 27 g Hamilton syringe was inserted parallel to the spine with rostral direction in order to reach the subarachnoid space. To ensure dura matter was breached, needle insertion resulted in a flick of the tail. The total volume was administered at a rate of 1 μl/s with 1‐min intervals after every 2 μl was dispensed. The needle remained in the intervertebral space after the delivery in order to ensure equilibrium without overflowing of cerebrospinal fluid. Ultimately, skin incision was secured with surgical sutures and mice were left to recover and wake up from anesthesia on a warm pad, ad libitum.

### 2.5 BrdU administration

In order to monitor proliferating cells, bromodeoxyuridine (BrdU), a thymidine analog which integrates into the DNA of dividing cells during S‐phase, was used [26]. On 50 dpi which marks the point of mice sacrifice (endpoint), four doses of BrdU were administrated to each animal (60 mg/kg of animal). After the animals were weighed, precise amount of BrdU was diluted in 1x PBS and the solution's pH was adjusted to 7.2–7.4. Each animal was injected i.p. with 500 μl BrdU every 2 h for four consecutive times and an hour after the last BrdU injection, mice were euthanized and perfused.

### 2.6 Tissue harvesting and processing

At 50 dpi endpoint, mice were euthanized and tissues (brains, spinal cords, lymph nodes, and spleens) were collected. Tissues for molecular analysis were quickly removed, snap‐frozen in liquid nitrogen, and stored at −80^ο^C until further processing could occur. Animals used for histopathology were transcardially perfused using PBS, followed by 4% paraformaldehyde in PBS (4% PFA) for approximately 5 min. Brain and spinal cords were carefully collected with entrenching meninges preserved as much as possible, post‐fixed in 4% PFA overnight at 4°C, and further processed for sagittal/longitudinal or transverse‐plane paraffin sectioning. Animals used for transmission electron microscopy (TEM), were transcardially perfused with 4% PFA containing 0.2% glutaraldehyde in 0.1 M PBS, post‐fixed for 1 h in 4% PFA, and immersed in 0.1 M PBS until semi and ultrathin sectioning.

### 2.7 Histological stains and immunohistochemistry

Routine histology stains were applied to 10‐μm‐thick serial longitudinal sections; Luxol fast blue (LFB) counterstained with nuclear fast red and Bielschowsky silver impregnation to assess myelin and axon maintenance, respectively, with protocols previously described [27]. Additionally, immunohistochemistry (IHC) for myeloid (Microglia) and lymphoid lineage cells (T and B cells) was performed to appraise the immunological aspect of the disease. Briefly, sections were deparaffinized, hydrated, and endogenous peroxidase was blocked with 3% H_2_O_2_ in methanol. Antigen retrieval was achieved using citrate buffer (pH = 6), followed by a 10% fetal bovine serum blocking buffer in PBS. Sections were incubated overnight (O/N) with Ionized calcium‐binding adaptor molecule 1 (rabbit Iba1; 019‐19741, Wako, 1:1000), rabbit CD3e (PAB9003, Abnova, 1:200), and rabbit B220 (BS‐4818R, Bioss, 1:200) primary antibodies and the IHC reaction was visualized with the EnVision^+^ System‐HRP Kit (DakoCytomation) for Iba1, T and B cells, respectively. Lastly, positive cells were stained dark brown using 3,3’‐Diaminobenzidine (DAB) (D5637, Sigma) as chromogen, and sections were counterstained with hematoxylin (Sigma).

### 2.8 Triple immunofluorescence

Similar to IHC described above, immunofluorescent detection was implemented to the serial sections with appropriate combinations of the following primary antibodies and dilutions: rabbit GFP (ab3080, Millipore, 1:200), mouse GFP (a‐11120, Thermo, 1:100), rabbit GFP (ab290, Abcam, 1:1000), rabbit Caspase3 (AF835, R&D Systems, 1:100), rat BrdU (ab6326, Abcam, 1:800), mouse BrdU (Bu20a, Cell Signaling, 1:500), mouse Nestin (MAB353, Millipore, 1:50), rabbit PDGFRa (AB61219, Abcam, 1:200), rabbit NG2 (ab5320, Millipore, 1:50), mouse MBP (NE1018, Millipore, 1:100), mouse Olig2 (MABN50, Millipore, 1:200), rabbit Nogo‐A (AB5888, Millipore, 1:200), mouse BCAS1 (sc‐393808, Santa Cruz, 1:200), rabbit GFAP (G6171, Sigma, 1:100), rabbit DCX (ab18723, Abcam, 1:100), mouse NeuN (MAB377, Millipore, 1:200), rabbit Connexin29 (34–4200, Thermo, 1:50), mouse Connexin32 (MAB3069, Chemicon, 1:100), and rabbit Connexin47 (36–4700, Thermo, 1:100). Accordingly, the secondary antibodies and dilutions applied were the following: goat anti‐rabbit Alexa Fluor 488 (a11008, 1:500), goat anti‐mouse Alexa Fluor 488 (a11001, 1:500), goat anti‐rat Alexa Fluor 555 (a21434, 1:500), goat anti‐rabbit Alexa Fluor 647 (ab150079, 1:500), and goat anti‐mouse Alexa Fluor 647 (a21235, 1:500). Sections were then mounted with 4’,6‐Diamidino‐2‐Phenylindole (DAPI; D1306, Invitrogen) and slides were coverslipped.

### 2.9 Neuropathology and connexin evaluation

All cell counting (cells/mm^2^) was executed by two different, blind to the experiment investigators, on six areas per section (which contained GFP^+^ cells) spaced at least 50 μm apart. Only Dapi^+^ and BrdU^+^ nuclei were taken into account. Images were captured using an Axioplan‐2 optical/widefield fluorescent (Zeiss) while confocal images were captured with Nikon Eclipse Ti and were assembled in ImageJ (Fiji) software. Demyelination, axonal loss, and inflammatory cell evaluation were analytically described in our previous work [27]. Connexin immunoreactivity was quantified with Bitplane Imaris software (Oxford Instruments) from analysis of multidimensional microscopy datasets. 3D reconstruction and visualization of the cells and connexins were rendered through the program from multiple z stacks. GJ plaques (Cx32 and Cx47) were defined as a concentration of connexin signal with size limits between 0.1 and 1 μm^2^ [28]. The total number of connexin GJ plaques (Cx32 and Cx47) was measured in each image and then a ratio to the respective cell (BrdU^+^) was calculated, as previously described [29].

### 2.10 Transmission electron microscopy and axonal morphometry

Lumbosacral spinal cord segments were post‐fixed in 1% osmium tetroxide (OsO_4_) for 2 h at room temperature (RT) and O/N in 0.1 M sodium acetate at 4°C. They were then dehydrated through gradual ethanol (50%, 70%, 95%, and 100%) and incubated O/N at RT in propylene oxide/epoxy resin 1:1. Segments were left to polymerize for 48 h at 60°C in fresh‐made epon epoxy resin. Resin blocks were removed from oven and stored at RT until semithin (0.5–1 μm) and ultrathin (50–100 nm) sectioning using EM UC6 (Leica). Semithin sections were stained in 1% toluidine blue for 30 s and sections were observed under brightfield microscopy with x100 oil objective. Ultrathin sections were collected on silver 200‐square mesh grids, briefly stained with drops of 1% uranyl acetate followed by lead citrate (Reynolds stain) and imaged on JEM‐1400 (Jeol) with a CCD camera (Gatan). Morphometric analysis (intact, dystrophic, preserved/normal appearing, demyelinated, and remyelinated axons per mm^2^) and g‐ratio assessment (calculated as the diameter of the axon diameter divided by the diameter of the axon plus myelin sheath) were extrapolated from the micrographs.

### 2.11 Statistical analysis

Quantitative data were evaluated and statistically analyzed using the GraphPad Prism 8.0 software. Normality level was accessed using the Kolmogorov–Smirnov test. Parametric data were reviewed using either unpaired *t*‐test or the one‐way ANOVA (specifically for EAE progression, a two‐way ANOVA was performed) with Bonferroni's post hoc test, depending on the appropriate criteria. Non‐parametric data were analyzed using Mann–Whitney or the Kruskall–Wallis test followed by Dunn's multiple comparison test, likewise. The level of significance was set at *p* < 0.05 and all cell counting values were expressed as mean ± SE.

## 3 RESULTS

### 3.1 EAE chronic disability is attenuated in NPC‐transplanted animals

**FIGURE 1:**
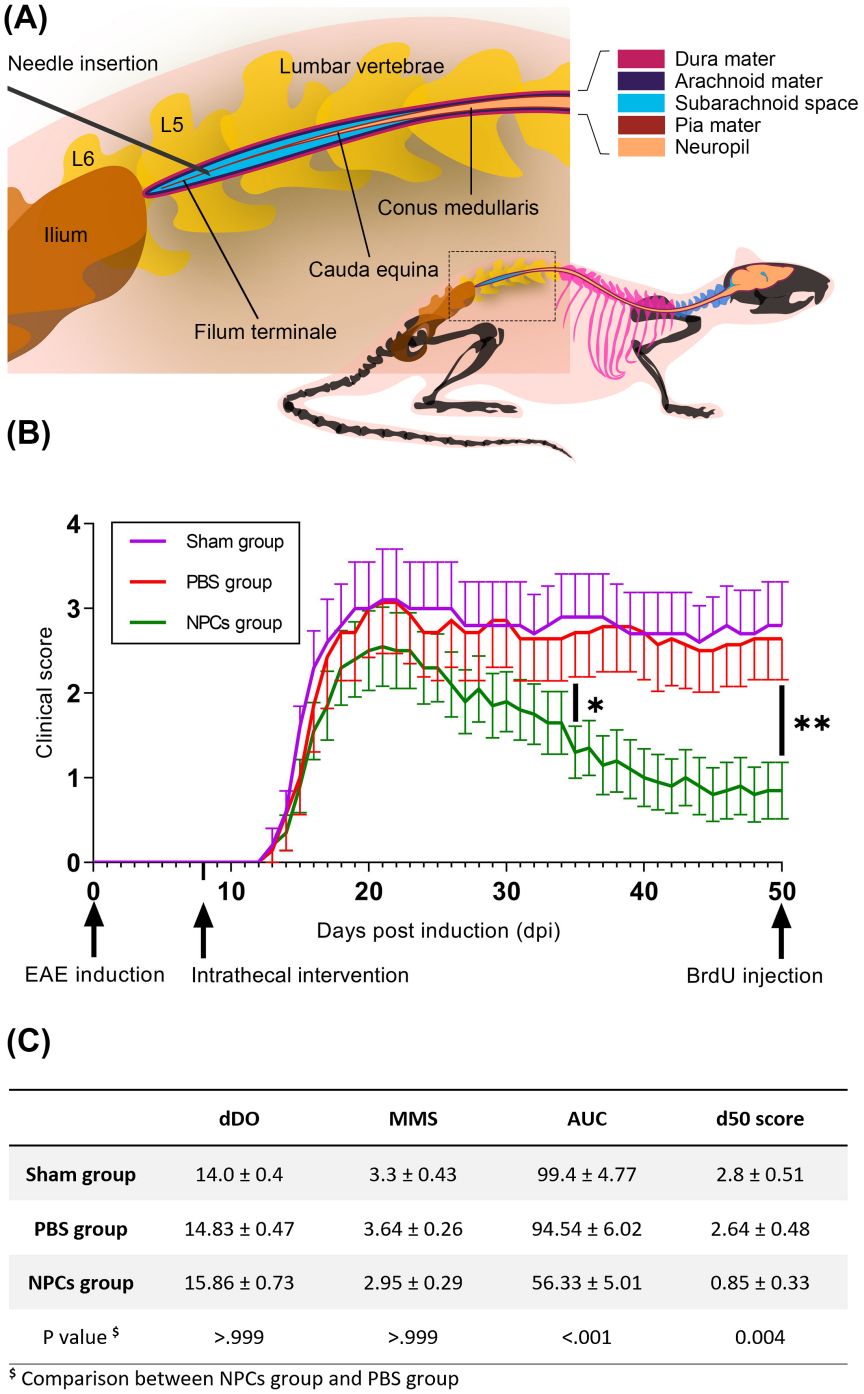
Schematic representation of intrathecal injection in the lumbar spine and clinical course of EAE groups. (A) Needle insertions targeted the cauda equina‐occupying lumbar cistern and were administered at the pre‐clinical phase, 8 days post‐EAE induction (8 dpi). (B) EAE clinical course of the three experimental groups. The first group (*N* = 13) received 10^6^ GFP^+^ NPCs in phosphate buffer (PBS), the second group (*N* = 10) received PBS alone and the third one served as the sham‐operated control group (*N* = 5) simulating the intervention. Three weeks after treatment (35 dpi) the NPCs group had a significantly lower clinical score (*p* < 0.05). (C) Determination of indexes such as mean day of disease onset (dDO), mean maximal score (MMS), mean area under the curve (AUC), and mean day 50 score (d50 score) in the three groups. The dDO and MMS did not manifest statistically significant differences (*p* > 0.999) between the three groups. At the endpoint, the NPCs group had a d50 score <1, which was significantly lower than the PBS group (*p* = 0.004). Data shown as mean ± SE, *p < 0.05, **p < 0.01, two‐way ANOVA

The experimental conditions of the lumbar intrathecal injection are illustrated graphically in Figure 1A. The intervention took place 8 days after the induction of EAE (Figure 1B) and the majority of these mice (*N* = 26) developed an acute relapse, starting on day 12, and peaking at day 20–22 post induction. We did not observe statistically significant differences in the mean day of disease onset (dDO) nor in the mean maximal score (MMS) (*p* > 0.999). The clinical outcome (overt paresis of the hind limbs) was maintained as the main phenotype of the sham‐operated and PBS‐treated groups. On the contrary, 3 weeks after treatment (35 dpi) and onwards, the NPC‐transplanted group displayed a lower score (d35 score; *p* < 0.05), reduced disability, and a marked recovery in their locomotion (mean area under the curve‐mAUC; *p* < 0.001). At the end of the follow‐up, when the animals exhibited a stable clinical score for 10 consecutive days, NPCs group had a mean score <1 (d50 score; *p* = 0.004) (Figure 1C). The sham‐operated animals displayed no statistically significant differences from the PBS group (*p* > 0.999), therefore only the PBS‐treated group was further analyzed.

### 3.2 NPCs contribute to the reduction of demyelination, axonal loss, and immune‐related cell populations

**FIGURE 2:**
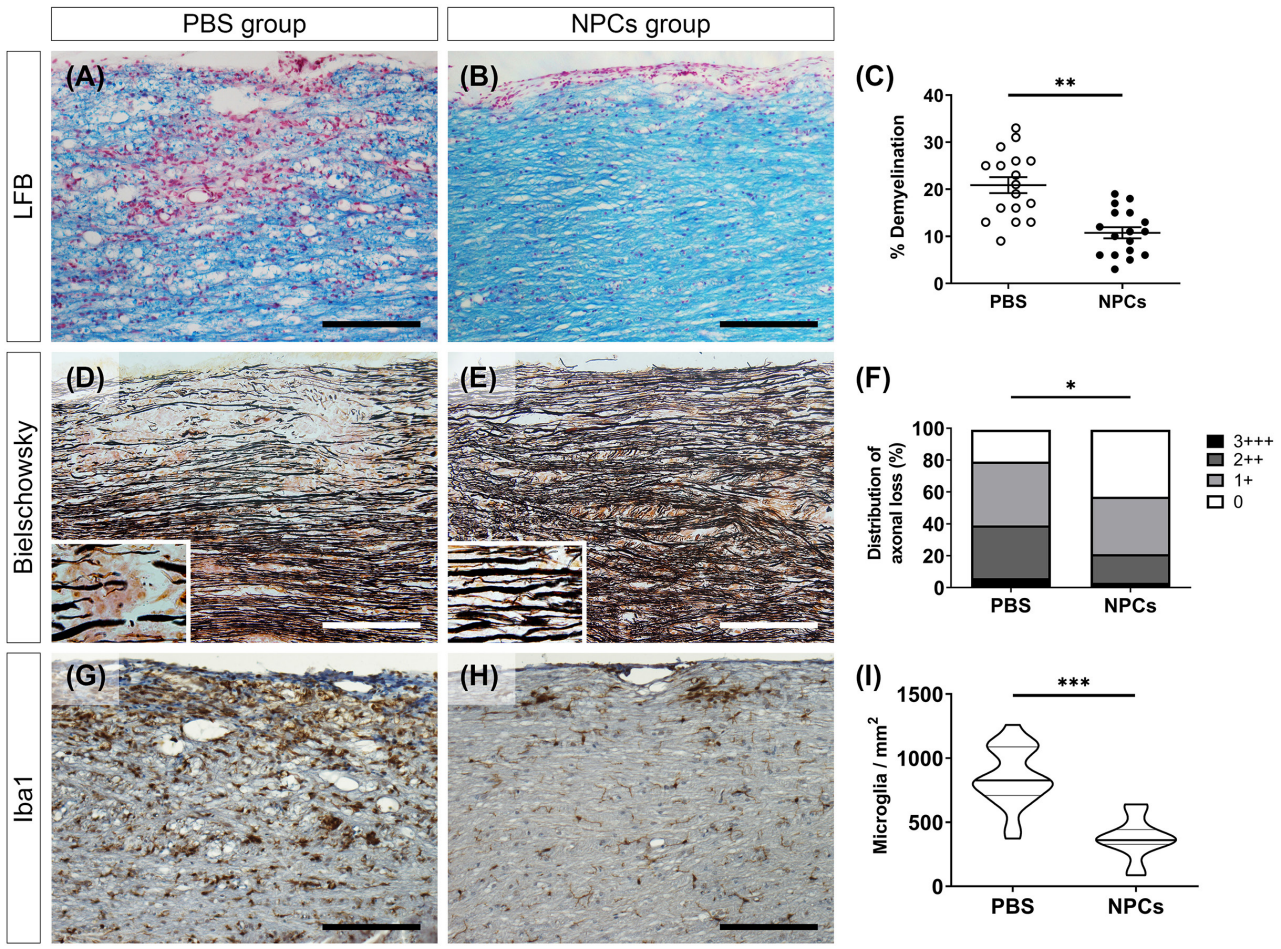
Extent of demyelination, axonopathy, and microglia activation in the lumbosacral area. (A, B) Longitudinal spinal cord sections stained with LFB and (C) the percentage of demyelination (demyelinated/total white matter area) in the two main experimental groups, PBS‐treated and NPC‐transplanted mice. Demyelination was significantly lower in the NPCs group compared to the PBS group (unpaired *t*‐test; *p* < 0.01). (D, E) Longitudinal spinal cord section stained with Bielschowsky's silver staining and (F) the percentage of axonal loss in PBS and NPCs groups, respectively. The severity of the damage was calculated and displayed as 0 = normal/even silver stain throughout the white matter; 1 = small spurious areas in the white matter that lack silver stain; 2 = small, but frequent, areas in the white matter that lack silver stain and 3 = extensive loss of silver stain throughout the white matter. The percentage of axonopathy was higher in the PBS group (chi‐square; *p* < 0.05). (G, H) Longitudinal spinal cord sections stained with Iba1 IHC and (I) measurements of Iba1^+^ cells per square millimeter around perivascular infiltrations from PBS and NPCs groups, respectively. Microglial cells were significantly higher in the PBS group compared to NPC‐transplanted mice (unpaired *t*‐test; *p* < 0.001). Scale bar = 100 μm

We firstly assessed the severity of chronic EAE on myelin and axons in the two experimental groups. LFB staining showed a significant decrease (*p* < 0.01) in demyelination of the NPCs group (10.76 ± 1.18%) compared to the PBS‐treated mice (20.88 ± 1.71%) (Figure 2A–C) and Bielschowsky silver staining showed that the percentage of damaged fibers was higher in the PBS group compared to the NPC‐transplanted mice (chi‐square; *p* < 0.05) (Figure 2D–F). We further analyzed the immunoreactivity for myeloid (activated and resting microglia), the main lymphoid lineage cells (T and B cells), and total perivascular infiltrates. Iba1 staining indicated that microglial cells were significantly higher in the PBS group (850.9 ± 51.10 cells/mm^2^) compared to the NPC‐transplanted mice (374.7 ± 34.14 cells/mm^2^) (Figure 2G–I). CD3e and B220 labeling showed a significant decrease of those immune populations in the NPCs group along with a concomitant decrease in total infiltrates (Figure S1A–C). Notably, the inflammatory areas in the transplanted mice were correlated with the location of the GFP^+^ cells, while showing a tendency to decline, especially in the lower white matter tracts (Figure S1D,E). It is evident from the data that the exogenously administered NPCs demonstrated potential immunomodulatory effects as previously proposed, associated with the protection of myelin and axonal integrity.

### 3.3 Transplanted cells were viable 50 dpi and exhibited efficient migration in the impaired CNS milieu

**FIGURE 3:**
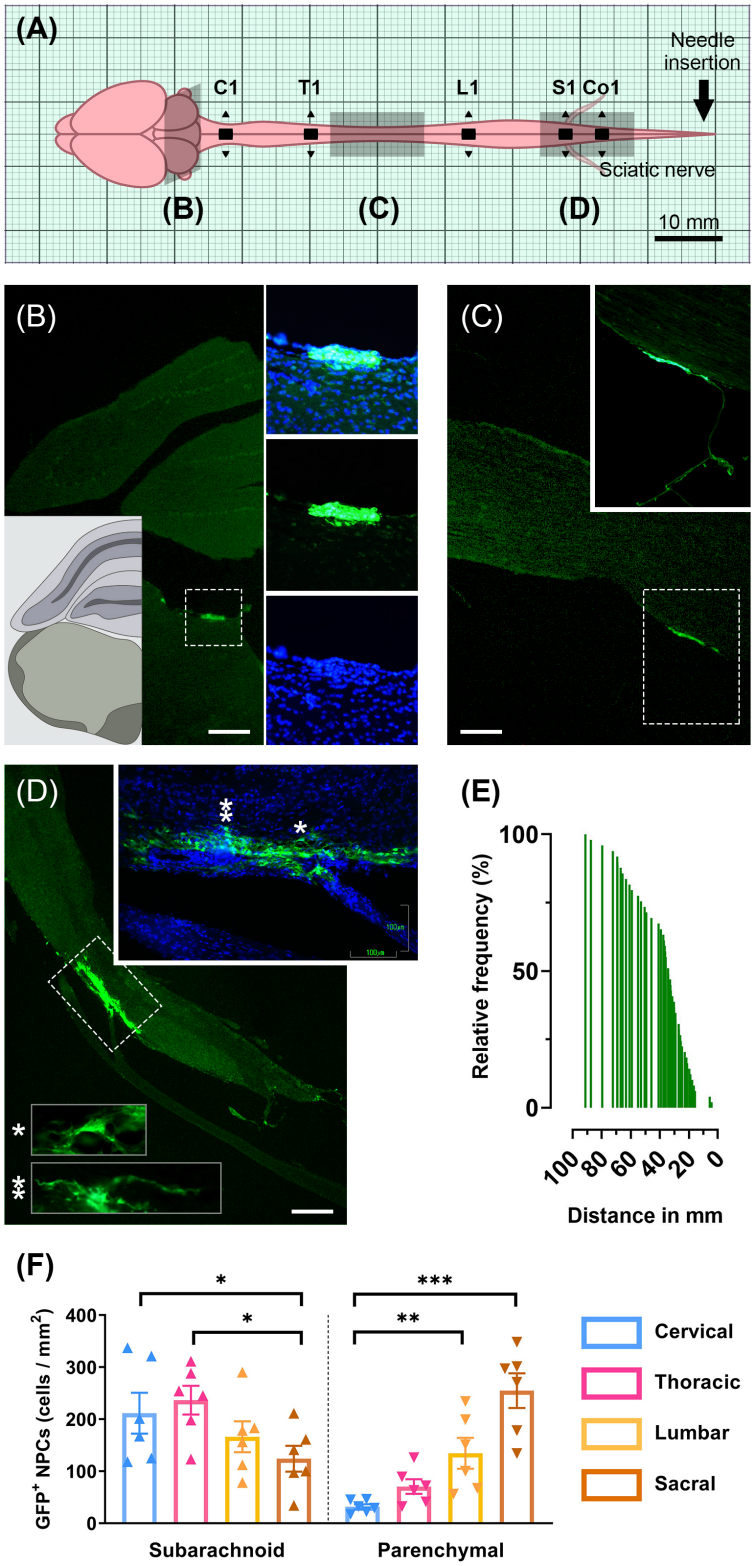
NPC migration patterns within the CNS. (A) GFP^+^ NPCs were identified in various subarachnoid areas of brain and spinal cord at the endpoint (50 dpi). (B) Cells found in brain and (C) upper / mid spinal cord regions potentially chemoattracted by the sparse lesions and remained clustered in the subarachnoid space. (D) GFP^+^ NPCs appeared more differentiated and integrated with the host parenchyma in lower spinal cord regions, where seemingly more lesions populated the lumbosacral fascicles. Asterisks correspond to cells in the high‐power field insets. (E) The majority of NPCs (~65%) found to occupy spinal cord regions between 20 and 40mm from the needle insertion site. (F) Two subpopulations of NPCs were determined. The ones that remained closer to perivascular infiltrates of the meninges displaying an undifferentiated phenotype (subarachnoid NPCs) as well as those that had migrated in the parenchyma exhibiting a more differentiated branched morphology (parenchymal NPCs). The first subpopulation prevailed in the cervicothoracic regions compared to lumbosacral (one‐way ANOVA; *p* < 0.05), whereas the second one was predominant in the lumbar and sacral funiculi. Data shown as mean ± SE, *p < 0.05, **p < 0.01, ***p < 0.001. Scale bar = 250 μm

At the disease endpoint, transplanted cells were distributed throughout the subarachnoid space of spinal cord (from the sacral to cervical regions) along with brain ventricular and subarachnoid regions (Figure 3A–D). No caspase3^+^ cells were found in those areas at the end of the follow‐up. Remarkably, NPCs were not detected in peripheral or any secondary lymphoid organs studied (data not shown). Starting from the injection site, the mean distance of transplanted NPC migration was 39.68 ± 2.93 mm (min 4.22 and max 91.13 mm). About 65% of all the cells were mobilized between 20 and 40 mm from the injection site which delineates the entire lumbosacral region (Figure 3E). Grafted NPCs exhibited two forms of clustering; the ones that lodged in close proximity to perivascular infiltrates of pial meninges that retained a seemingly undifferentiated phenotype (subarachnoid NPCs) and the ones that migrated in the parenchymal neuropil showcasing a developed, ramified morphology (parenchymal NPCs). Subarachnoid NPCs were significantly more prevalent in the cervicothoracic regions compared to lumbosacral (*p* < 0.05), while parenchymal NPCs numbers were found significantly higher in lumbar (134.5 ± 29.58 cells/mm^2^) and sacral spinal cord segments (254.8 ± 33.34 cells/mm^2^) (Figure 3F).

### 3.4 NPCs acquire oligodendroglia lineage characteristics and further participate in host tissue oligodendrocytic enrichment

**FIGURE 4:**
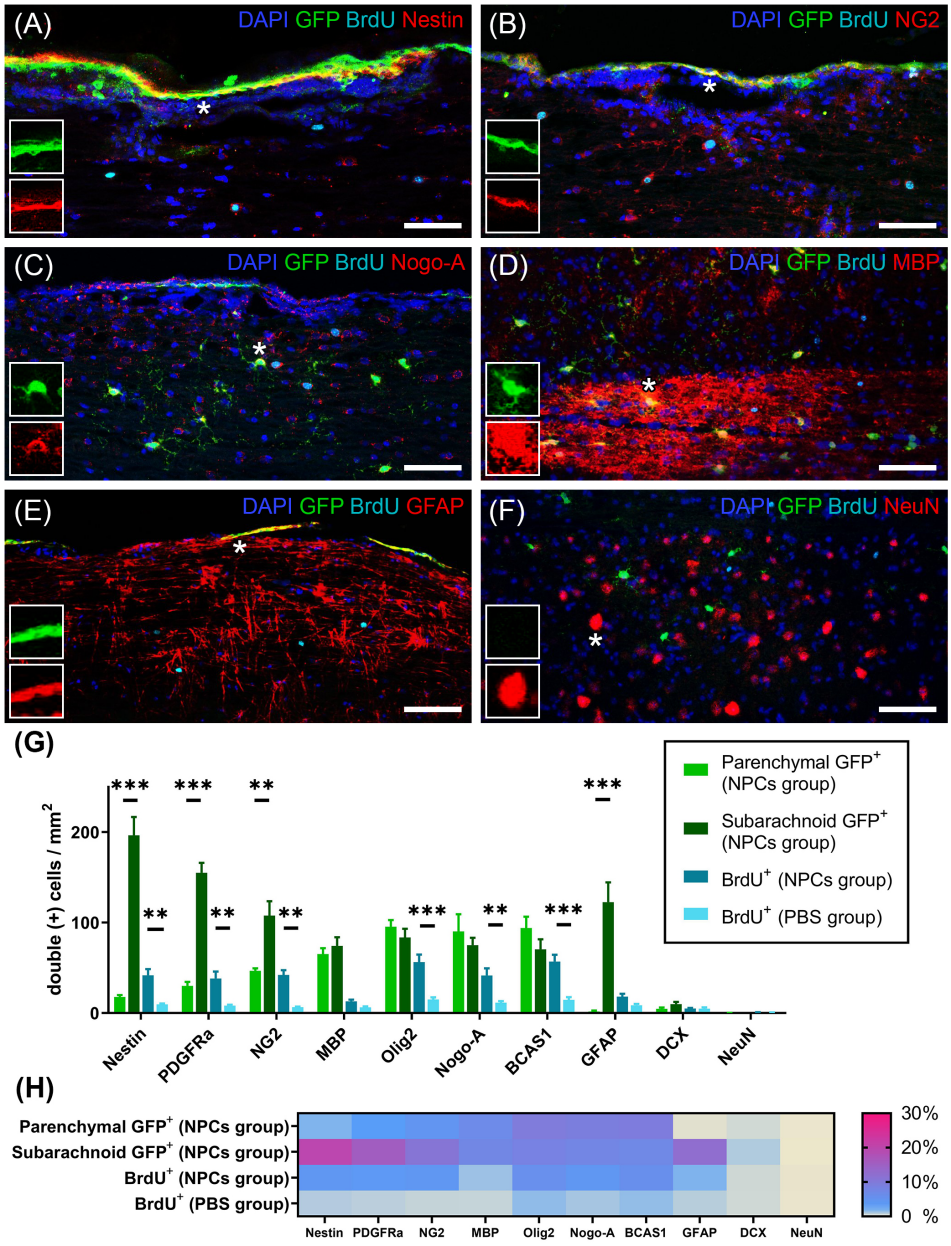
Differentiation profile of transplanted and endogenous cells in chronic EAE lesions. (A–F) Representative longitudinal lumbosacral spinal cord sections triple‐immunolabeled for GFP (green), BrdU (cyan) along with miscellaneous glial and neuronal markers (red). Cell nuclei were counterstained with DAPI (blue). Scale bar = 50 μm. (G) Double positive cells (GFP and marker, BrdU and marker) were measured and expressed as cells per square millimeter for each individual marker. GFP^+^ cells in the NPCs group were subdivided into parenchymal (light green) and subarachnoid (dark green) cell populations. Subarachnoid NPCs remained less differentiated, expressing higher levels of Nestin, PDGFRa, NG2, and GFAP compared to parenchymal cells (unpaired *t*‐test; **p < 0.01, ***p < 0.001). Additionally, BrdU^+^ cells were increased in the NPCs group when compared with respective areas of the PBS group (unpaired *t*‐test; **p < 0.01, ***p < 0.001). (H) Percentage expression of all markers. Mature oligodendrocyte markers (MBP, Olig2, Nogo‐A, and BCAS1) were expressed in both parenchymal and subarachnoid GFP^+^ cells, whereas GFAP was significantly higher in the subarachnoid population. The BrdU^+^ cells exhibited higher immunoreactivity in precursor and oligodendrocyte‐related cells, in contrast to very low percentages of GFAP^+^ astrocytes, DCX^+^ neuroblasts, and NeuN^+^ neurons

Next, we investigated the differentiation profile of the transplanted cells while investigating their effect on the endogenous cell proliferation in the respective rehabilitated areas. A total of ten macroglial and neuronal antigen markers were interrogated in sagittal lumbosacral spinal cord sections (Figure 4A–F, Figure S2) identifying that NPCs followed a spatial phenotypic variability. Subarachnoid NPCs remained less differentiated (196.6 ± 20.08 cells/mm^2^ Nestin^+^, 155.2 ± 11.03 cells/mm^2^ PDGFRa^+^, and 107.8 ± 15.88 cells/mm^2^ NG2^+^) and were significantly higher compared to the parenchymal cells (17.8 ± 2.05 cells/mm^2^ Nestin^+^; *p* < 0.001, 30 ± 4.57 cells/mm^2^ PDGFRa^+^; *p* < 0.001, and 46.8 ± 2.7 cells/mm^2^ NG2^+^; *p* < 0.01, Figure 4G). GFAP^+^‐differentiated NPCs were also significantly different (subarachnoid 122.6 ± 21.85 cells/mm^2^ vs. parenchymal 1.8 ± 0.97 cells/mm^2^; *p* < 0.001). On the contrary, mature oligodendocytic markers such as MBP, Olig2, Nogo‐A, and BCAS1 exhibited high percentages (15%–25%) in both forms (Figure 4H). Noteworthy is the low percentages of DCX^+^ and almost nonexistent NeuN^+^ NPCs among both groups.

The BrdU incorporation was efficient to mark the proliferative cells at the experimental end point as the cells underwent DNA replication (S phase) expressing the majority of the markers therein used (Figure 4A–F, Figure S2). Throughout all cellular phenotypic comparisons, BrdU^+^ cells that were found in close vicinity to GFP^+^ NPC‐derived cells, were markedly increased in the NPCs group versus the PBS group (Nestin, PDGFRa, NG2, Nogo‐A; *p* < 0.01 and Olig2, BCAS1; *p* < 0.001, Figure 4G). The highest percentages of labeled cells were documented in the precursor and oligodendrocyte‐related cell populations (4.2% NG2^+^, 5.6% Olig2^+^, 4.1% Nogo‐A^+^, 5.7% BCAS1^+^), while the lowest were in the GFAP^+^ (1.8%) and neuronal cell populations (<0.5%) (Figure 4H). Surprisingly, we could not identify GFP^+^/BrdU^+^ in the particular loci. Taken together, co‐expression of BrdU^+^ cells with several oligodendrocyte differentiation markers suggests that in addition to transplanted GFP^+^ NPCs, the endogenous precursor cell activation is also expanded by proliferation, potentially remyelinating lesions in the spinal cord.

### 3.5 BCAS1 and Olig2 subpopulations are reinforcing host myelination by exhibiting direct interaction through oligodendrocytic connexins

**FIGURE 5:**
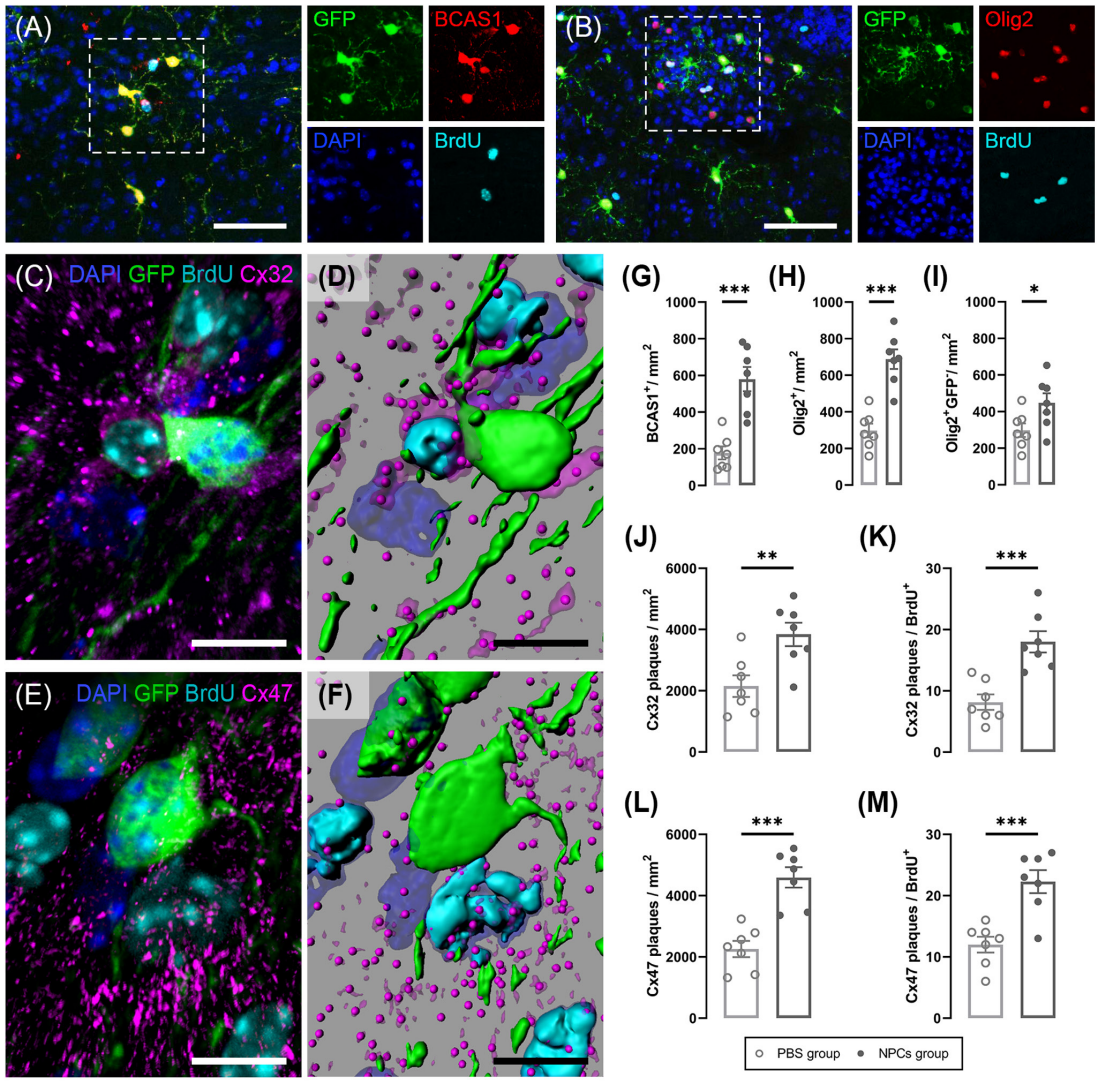
NPC integration evaluated with oligodendrocyte connexin expression. (A, B) Representative longitudinal lumbosacral spinal cord sections triple‐immunolabeled for GFP (green), BrdU (cyan) along with BCAS1 and Olig2 (red), respectively. Cell nuclei were counterstained with DAPI (blue). Scale bar = 50 μm. (C, E) Triple immunofluorescence and (D, F) 3D reconstruction of GFP (green), BrdU (cyan) along with connexin32 and connexin47 (magenta) markers showcasing increased Cx32 and Cx47 signal around GFP^+^ cells. Scale bar = 10 μm. (G, H) Parenchymal BCAS1^+^ and Olig2^+^ NPCs were significantly higher in the NPC‐ versus PBS‐treated mice (unpaired *t*‐test; *p* < 0.001). (I) BrdU^+^/Olig2^+^/GFP^−^ cells accounting for the newly proliferated endogenous OPCs were considerably increased in the NPC‐transplanted group. (J, L) Cx32 and Cx47 plaques per square millimeter and (K, M) per BrdU^+^ cell, were also twofold higher in NPCs group compared to PBS group, respectively. Data shown as mean ± SE, **p < 0.01, ***p < 0.001

The most consistent antigen markers that we observed among the parenchymal NPCs and recipient tissue were BCAS1^+^ and the transcription factor Olig2^+^ (Figure 5A,B). BCAS1^+^ oligodendrocytes were significantly higher in the NPC‐transplanted group compared to the PBS‐treated group (579.7 ± 66.2 cells/mm^2^ vs. 177.4 ± 35.37 cells/mm^2^; *p* < 0.001). These results were also recapitulated for the total Olig2^+^ oligodendrocytes (687.6 ± 53.35 cells/mm^2^ vs. 298.3 ± 37.41 cells/mm^2^; *p* < 0.001) but also, and most importantly, for the BrdU^+^/Olig2^+^/GFP^−^ oligodendrocytes (447.9 ± 52.60 cells/mm^2^ vs. 298.3 ± 37.41 cells/mm^2^; *p* < 0.05) accounting for the newly proliferated endogenous OPCs that were effectively activated by the NPCs transplantation (Figure 5G–I). This finding led us to additionally investigate all the oligodendrocytic‐forming gap junctions, namely Cx29, Cx32, and Cx47 in an attempt to further characterize the proliferative, BrdU^+^ cells. We could not detect any labeling for Cx29. On the other hand, Cx32 and Cx47 plaques were highly increased in GFP^+^ areas (Figure 5C–F) and significantly higher (2‐fold) per mm^2^ and per BrdU^+^ cell when compared with the PBS‐treated group (Figure 5J–M). Therefore, these demonstrate a well‐established oligodendrocyte integration of NPCs.

### 3.6 Transplanted NPCs promotes remyelination of small‐caliber nerve fibers

**FIGURE 6:**
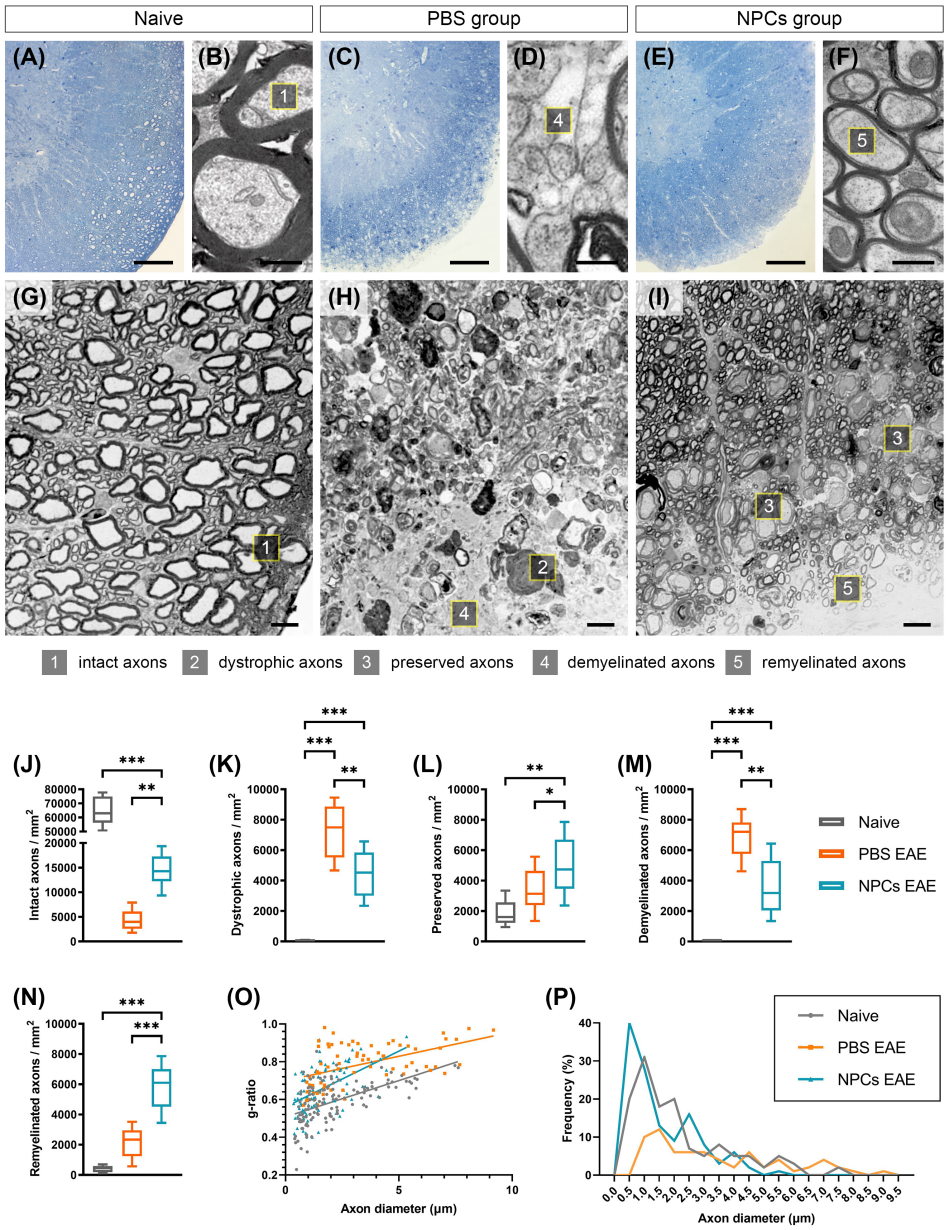
Axonal morphometry and validation of remyelination. (A, C, E) Lumbosacral semithin (0.5–1 μm) sections from naive, PBS and NPCs groups, stained with toluidine blue and observed under high‐power brightfield microscopy. Scale bar = 100 μm. (B, D, F) Lumbosacral ultrathin (50–100 nm) sections from naive, PBS, and NPCs groups were observed in TEM, respectively. Scale bar = 0.5 μm. (G, J) Naive white matter funiculi consisted predominately of intact, large‐diameter axons. (H, K, M) The PBS group exhibited extensive subpial lesions with more dystrophic and demyelinated axons than NPCs group (one‐way ANOVA; *p* < 0.01). (I, L, N) The NPCs group manifested many larger diameter, normal appearing (seemingly preserved) axons, and high levels of remyelinated fibers (*p* < 0.001). (O) The mean g‐ratio, which was 0.59 in naïve mice, increased to 0.65 in NPC‐transplanted mice. (P) Diagram representing the frequency percentage in relation to the axon diameter. The NPCs group consisted of about 40% small‐caliber fibers. Data shown as mean ± SE, *p < 0.05, **p < 0.01, ***p < 0.001. Scale bar in G–I = 10 μm

Since we identified these oligodendrocyte populations, we investigated their reparative capacity that correlated with neurological improvement. More specifically, we utilized osmium‐fixed, epon‐embedded sample tissue from the ventrolateral funiculi of the three groups (naïve, PBS, NPCs) in semithin (Figure 6A,C,E) and ultrathin sections using transmission electron microscopy (Figure 6B,D,F). As expected, the naïve tissue contained exclusively intact axons (Figure 6G,J; 64,519 ± 3029 axons/mm^2^) with a big diameter and a mean g‐ratio of 0.59 (Figure 6O). The PBS‐treated mice exhibited huge subpial lesions within the ventrolateral funiculi (Figure 6H) with extensive dystrophic/degenerated (Figure 6K; 7329 ± 535.3 axons/mm^2^) and demyelinated axons (Figure 6M; 6928 ± 406 axons/mm^2^).

Conversely, the NPC‐transplanted group showed a significantly lower amount of both dystrophic (Figure 6K; 4459 ± 499.8 axons/mm^2^) and demyelinated axons (Figure 6M; 3584 ± 566.7 axons/mm^2^) when compared with the PBS‐treated group (*p* < 0.01). A considerable proportion of normal appearing, preserved axons were present in the proximal spinal cord white matter regions (Figure 6L; 4994 ± 547 axons/mm^2^). These axons were much bigger in diameter than the remyelinated ones (Figure 6I), with a typical dysregulated myelin sheath, found further away from the subpial surface. Lastly, the remyelinated fibers in this group were significantly higher than the PBS‐treated group (Figure 6N; 5928 ± 460.3 vs. 2123 ± 316.6; *p* < 0.001) with a g‐ratio of 0.65 versus naïve 0.59; *p* < 0.001 (Figure 6O) and a relatively small mean axon diameter (40% with 0.5 μm; Figure 6P). The latter results denote that the transplanted NPCs helped toward the appearance of those newly myelinated small‐caliber fibers in the areas that have been previously subjected to demyelination.

## 4 DISCUSSION

The degenerative mechanisms of many neurological diseases often impede the CNS, up to a point where its endogenous mechanisms are incapable of repairing the cumulative lesions. Therefore, transplanted NPCs have appeared in the stem cell research spotlight as a promising therapeutic solution for severe neurological disorders [30]. These potent cells have been investigated not only for their immunomodulatory competence but also for their ability to replace damaged cells like oligodendrocytes and promote axonal remyelination [30, 31]. There are couple animal models of MS [32] to investigate their potential and EAE is one of them, although it is considered by some to be an acute immune‐mediated axonopathy without any remyelination, while others have suggested a sufficient element of demyelination, allowing potential remyelination to occur [32, 33]. However, one clinically relevant question that remains unresolved is determining the optimal routes of administration and the optimal conditions for the least invasive and damaging cell delivery method [34].

Our study demonstrates that grafted NPCs in the form of neurospheres administered near a prominent, anticipated demyelination in the lumbosacral area, promoted neurological recovery exhibited by abrogated hind limb paralysis in EAE‐induced mice. The clinical amelioration was not observed in a previous ICV transplantation of NPCs developed under same experimental conditions, although the beneficial histopathological hallmarks, were recapitulated in the cc [10]. This discrepancy might have been attributed to the immunomodulatory effects these cells exert in the immunocompromised demyelinated CNS milieu [9, 30], but the localized, physical interaction triggered proliferation and accelerated the reparative process. Hence, apart from the ICV [5], intravenous [16] or cisterna magna [17, 19], the lumbar cisterna has emerged as an equally suitable area for injection of NPCs as have been predominantly used to treat experimental spinal cord injury (SCI) [34, 35, 36, 37].

We further appraised the viability of transplanted NPCs, migratory pattern, and differentiation. At 50dpi, NPCs were found to populate the CNS in a density gradient, identifying less cells throughout brain areas and more localized to the spinal cord. This finding can be attributed to neurotrophic and cytokine signals arising from ectopic atypical perivascular niches that are the chemoattractants for these cells, as that has been successfully replicated in vitro [22, 25]. Furthermore, transplanted cells exhibited two main spatial patterns; the subarachnoid and the parenchymal subpopulations. As the names imply, the subarachnoid clusters were scattered in the superficial leptomeningeal areas and remained less differentiated (mainly nestin^+^ and GFAP^+^), whereas parenchymal NPCs, noticeably differentiated, traversed the neuropil expressing predominantly oligodendrocyte markers. Among those, MBP, a structural protein that participates in myelin sheath formation [38], as well as Olig2 transcription factor [4, 39], Nogo‐A, and BCAS1 are significant myelinating oligodendrocyte markers found consistently increased in chronic EAE [23] and at the edges of demyelinating MS lesions [40, 41].

In the context of determining the transplantation effects on endogenous precursor cell proliferation, we detected the DNA synthesis with BrdU labeling [26]. Nuclear Ki67, a protein of G1, S, G2, and M cell cycle phase, albeit it has been used in relevant case scenarios, its application was problematic due to localization, half‐life, and detection [42, 43]. Likewise, proliferating cell nuclear antigen (PCNA), a protein of DNA polymerases that is associated with G1 and S phases, has also raised concerns because it can be found in non‐proliferating cells as it participates in DNA repair mechanisms [43]. In our results no BrdU^+^/GFP^+^ cells were identified, indicating that the NPCs have become post‐mitotic and BrdU^+^ cells were of endogenous origin. On the other hand, more progenitors and mature forms of oligodendrocytes were found to be BrdU^+^ in the NPC‐transplanted group suggesting that the affected milieu harbor an innate ability of bone fide oligodendrogenesis as seen in a timely fashion developmental need [6, 44]. Non‐detectable levels of DCX and NeuN implicated that oligodendrocyte lineage cells were predominant within the respective lesion.

NPCs transplantation resulted in higher levels of progenitors such as NG2^+^ as a response to demyelination [3], but mostly Olig2^+^ and BCAS1^+^ populations, which presumably define early myelinating oligodendrocytes [4, 41]. In order to quantify how strong the integration of these newly formed oligodendrocytes was, we evaluated the distributions of the gap junction proteins Cx29, Cx32, and Cx47 [45]. The localization of Cx32 has been identified throughout myelin connections with large diameter fibers [46]. On the other hand, Cx47 expression is more perikaryon‐based, developmentally expressed at an earlier timeframe than Cx32 [47] and has been detected on both myelinating oligodendrocytes and OPCs [28, 48]. Lastly, the inadequate Cx29 signal we received may be attributed to the fact that it only forms hemichannels on small fibers of optic nerve and cc, where Cx32 is commonly absent [45]. Interestingly, mice with Cx32 or Cx47 deficiency can be more susceptible to EAE and exhibit prominent demyelination and microglia activation [29, 48].

Moving further toward a staple, functional and structural index of remyelination, we computed the g‐ratio which relates the myelin volume with regards to the fiber volume fraction [49]. The morphometric analysis enabled us to identify normal and normal appearing (preserved) axons, dystrophic, with onion‐like appearance degenerated fibers, completely denuded axons (demyelinated), and small‐caliber fibers with thin myelin sheaths mainly found in the most superficial tracts of white matter [50]. We postulate that the proliferative host oligodendrocytes, the parenchymal NPCs, and even up to a degree the subarachnoid NPCs—mainly because of the spatial continuity to subpial layers—shifted the g‐ratio, which practically translates to clinical rehabilitation [51, 52]. This location‐based neuroprotective effect has been also achieved in experimental SCI [36]. However, it remains elusive how the immunological changes during the relapse phase of EAE affected the NPCs contribution to myelin preservation.

## 5 CONCLUSIONS

In conclusion, we report that a single intrathecal lumbar spine administration of NPCs can deliver a viable cell load that is maintained up to 50dpi, limiting effectively the expected oligodendrocytic depletion and chronic demyelination of EAE. This is the first study that delineates the roles of NPCs specifically on host oligodendrogenesis (through BrdU^+^/Olig2^+^/GFP^−^ cells) and in vivo oligodendrocyte proliferation in the demyelinating milieu. Our results suggest that this was potentiated through a direct, in situ preservation of the oligodendrocyte chemical coupling and also through remyelination of subpial white matter funiculi, in a model which is otherwise devoid of such capability. The transplantation paradigm implemented hereunder, epitomizes the first step toward a future stem cell‐based MS therapy where genome editing could potentially amplify the neuroprotective effect of these cells and circumvent any clinical relapse a priori.

## CONFLICT OF INTEREST

The authors declare that they have no competing interests.

## AUTHOR CONTRIBUTIONS

Paschalis Theotokis carried out the EAE and transplantation experiments, visualized the data, and wrote the manuscript. Evangelia Kesidou performed the cell culture and contributed to the design and implementation of the research. Dimitra Mitsiadou collected and analyzed the BrdU data. Steven Petratos provided resources and edited the manuscript. Olympia Damianidou assisted with the data curation. Marina Boziki arranged administrative tasks and critically reviewed the manuscript. Anastasia Chatzidimitriou assisted with funding acquisition and critically reviewed the manuscript. Nikolaos Grigoriadis conceptualized the idea and formulated the design of the study. All authors read and approved the final manuscript.

## ETHICS APPROVAL

All experimental procedures were conducted according to the institutional guidelines, in compliance with the Greek Regulations and the European Communities Council Directive of November 24, 1986 (86/609/EEC) for use of laboratory animals. Experimentation received approval from the veterinary medicines directorate (protocol number 211531/1490).

## Supporting information


**FIGURE S1** Alteration in immune cell populations after transplantation of NPCs. (A–C) Quantification of perivascular infiltrates, CD3^+^ and B220^+^ cells indicating the decrease of these specific lineages in the NPC‐treated group. (D, E) Correlation of the lesion area with the location of the GFP^+^ cells in the NPC‐transplanted group, demonstrating the decrease in the inflammatory extent, especially in the lower regions where GFP^+^ signal was predominant. Our results corroborate the notion that transplanted NPCs have an immunomodulatory effect while restricting the expected demyelination. Data shown as mean ± SE, **p < 0.01, ***p < 0.001. Scale bar = 350 μm

**FIGURE S2** Analytical NPCs differentiation profiling. (A–J) Representative longitudinal lumbosacral spinal cord sections displaying all the immunofluorescent channels for each individual marker used in this study; GFP (green), BrdU (cyan), miscellaneous glial, and neuronal markers (red). Nuclei were counterstained with DAPI (blue). Scale bar = 50 μm

## Data Availability

The datasets analyzed during the current study are available from the corresponding author on reasonable request.
