# Supplementary figures and images for: Lumbar spine intrathecal transplantation of neural precursor cells promotes oligodendrocyte proliferation in hot spots of chronic demyelination
Source: Brain Pathol. 2021 Nov 29;32(4):e13040. doi: 10.1111/bpa.13040 (PMC9245942; doi:10.1111/bpa.13040)

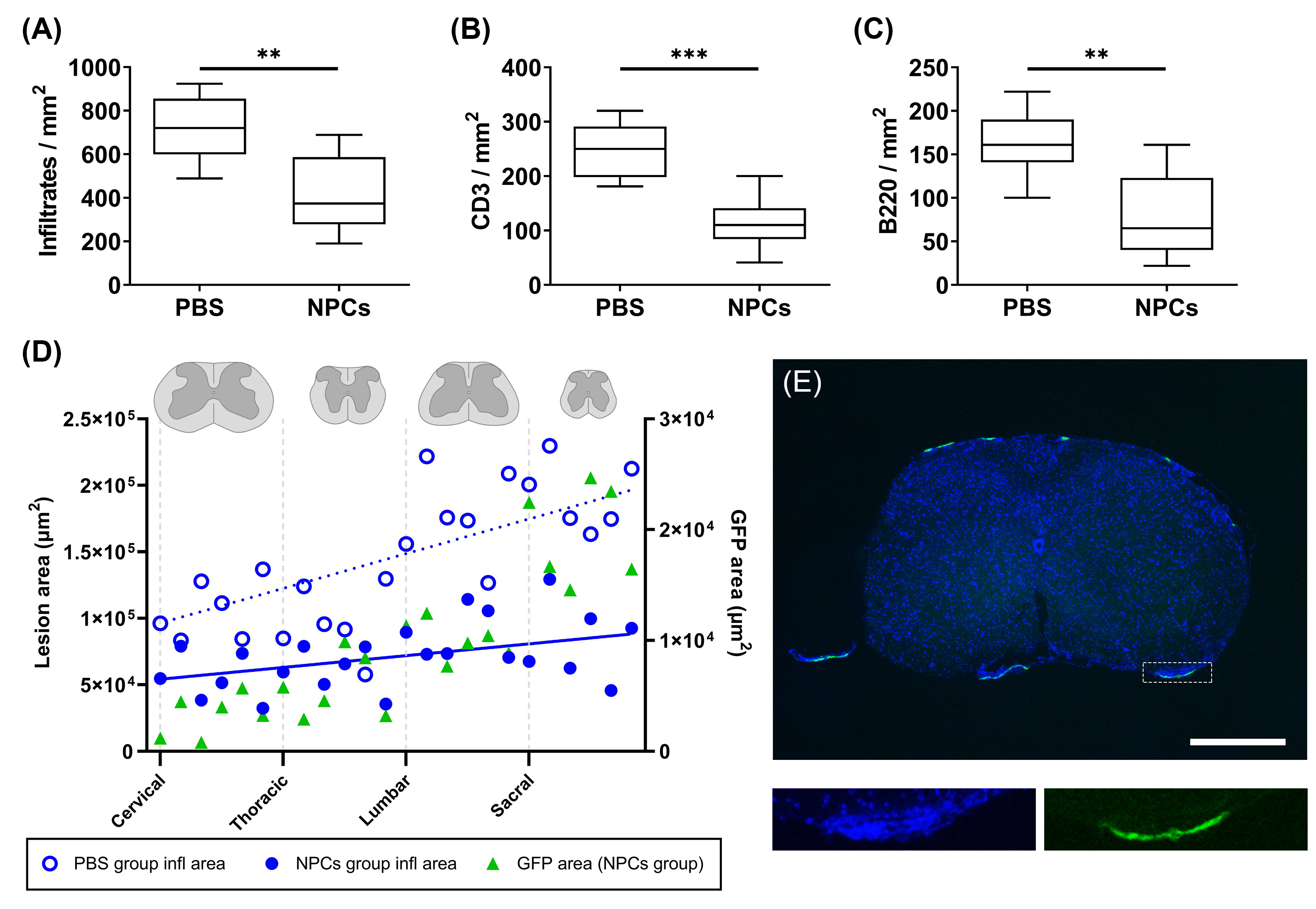

Supplement: Supplementary file 1 — FIGURE S1 Alteration in immune cell populations after transplantation of NPCs. (A–C) Quantification of perivascular infiltrates, CD3+ and B220+ cells indicating the decrease of these specific lineages in the NPC‐treated group. (D, E) Correlation of the lesion area with the location of the GFP+ cells in the NPC‐transplanted group, demonstrating the decrease in the inflammatory extent, especially in the lower regions where GFP+ signal was predominant. Our results corroborate the notion that transplanted NPCs have an immunomodulatory effect while restricting the expected demyelination. Data shown as mean ± SE, **p < 0.01, ***p < 0.001. Scale bar = 350 μm [file BPA-32-e13040-s001.jpg]

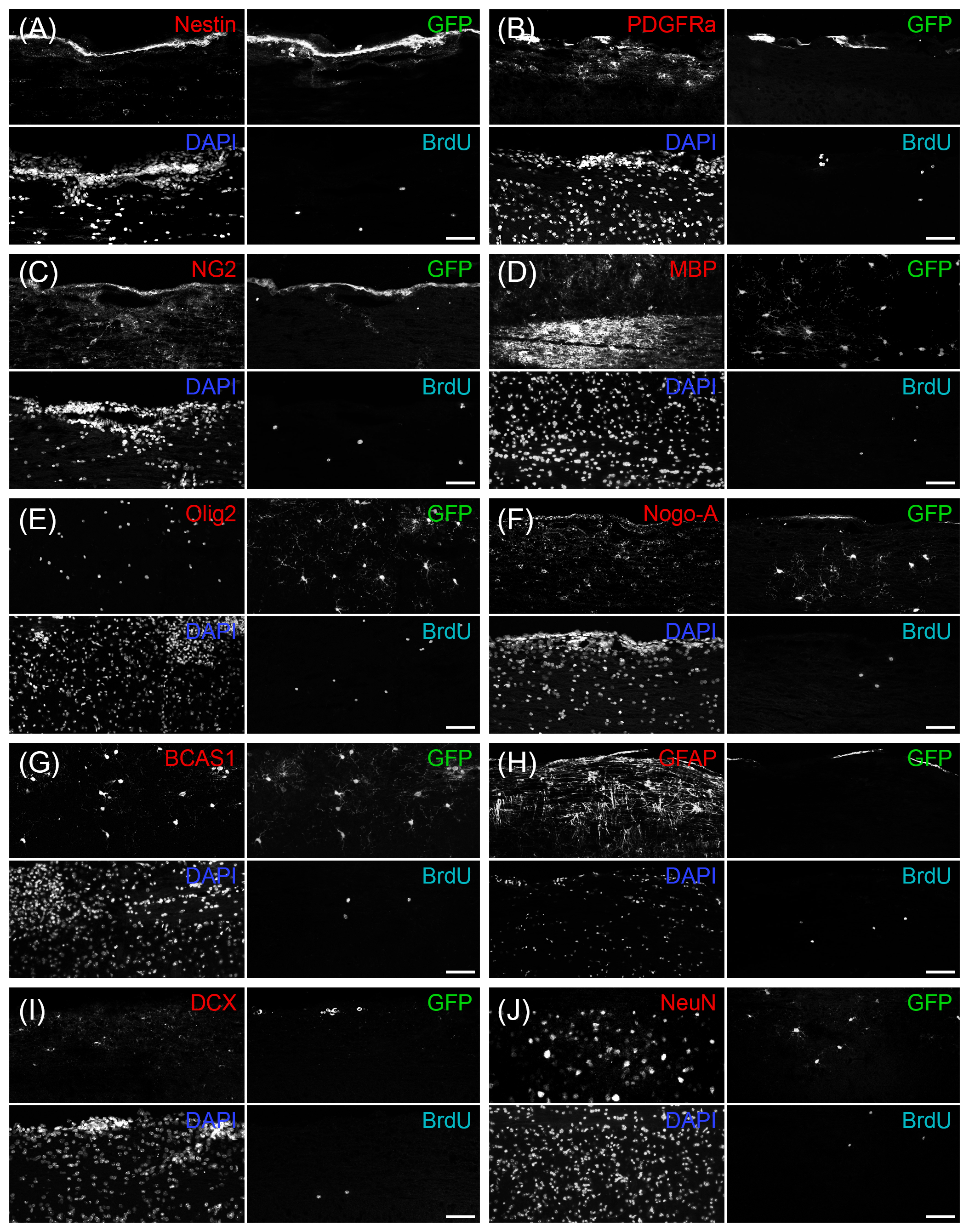

Supplement: Supplementary file 2 — FIGURE S2 Analytical NPCs differentiation profiling. (A–J) Representative longitudinal lumbosacral spinal cord sections displaying all the immunofluorescent channels for each individual marker used in this study; GFP (green), BrdU (cyan), miscellaneous glial, and neuronal markers (red). Nuclei were counterstained with DAPI (blue). Scale bar = 50 μm [file BPA-32-e13040-s002.jpg]
